# Oropharyngeal and Oral Cancer in Lung Cancer Patients: Do They Present a Worse Prognosis than Isolated Lung Cancer Patients?

**DOI:** 10.3390/cancers17111850

**Published:** 2025-05-31

**Authors:** Farzin Falahat Noushzady, Sonia Herrero Álvarez, Joaquín Calatayud Gastardi, Elena María Vara-Ameigeiras, Carlota Mazo Amorós, Irene Serrano-García, Florentino Hernando Trancho, José Ramón Jarabo Sarceda, Ana Maria Gómez Martínez

**Affiliations:** 1 Department of Surgery, School of Medicine, Complutense University of Madrid, Avda. Complutense, s/n, 28040 Madrid, Spainanamagom@ucm.es (A.M.G.M.); 2 Department of Biochemistry and Molecular Biology, School of Medicine, Complutense University of Madrid, Avda. Complutense, s/n, 28040 Madrid, Spain; 3 Unidad de Apoyo Metodológico a la Investigación, Instituto de Investigación Sanitaria del Hospital Clínico San Carlos (IdISSC), Hospital Clínico San Carlos, C/Profesor Martín Lagos s/n, 28040 Madrid, Spain

**Keywords:** multiple primary malignancies, second primary cancer, lung cancer, oropharyngeal cancer, survival rate, overall survival

## Abstract

**Background/objectives:** Second primary lung cancer frequently manifests in individuals who have survived head and neck cancer, with this occurrence often being attributed to shared risk factors. The objective of the present study is to compare the prognosis, in terms of survival rate, of patients who presented isolated bronchogenic carcinoma (BC) with that of patients who presented with a personal history of BC and associated oropharyngeal and oral cancer (OAOC). **Methods:** A retrospective longitudinal study was conducted, including all consecutive patients who underwent surgical resection with curative intent for BC in Hospital Clínico San Carlos (HCSC), Madrid, Spain, between December 1989 and December 2024. The survival rate was calculated and compared in two groups of patients: Group 1: 1594 patients with isolated BC and Group 2: 97 patients with BC and OAOC. **Results:** Group 2 did not show a significant difference in their 60-month survival rate in comparison to Group 1 (HR = 1.23, CI 95% 0.9–1.6) *p* = 0.14. But when comparing the 120-month survival rate, Group 1 showed a significantly higher survival rate (36.4%, CI 95% 33.9–39%) compared to Group 2, (25.54%, CI 95% 17.78–36.7%) HR= 1.28 (CI 95% 1–1.6), *p* = 0.04. Disease-free survival showed a non-significant trend of greater severity among patients with a previous history of OAOC. **Conclusions**: Lung cancer patients who presented with OAOC had worse overall survival compared to patients who presented with isolated lung cancer, and a significant difference was observed at 120 months of follow-up.

## Simple Summary

The incidence of Multiple Primary Cancers has increased in the past decade. Each cancer has its own prognosis, and this plays a vital role in patient management and clinical decision-making. In case of Multiple Primary Cancers, the estimation of their incidence and survival rate are not as simple as in the cases of single primary cancers, since two or more malignancies play part. The objective of this study is to draw a comparison between the survival rates of patients diagnosed with bronchogenic carcinoma who underwent radical surgical treatment and those who underwent the same treatment while having a personal history of oropharyngeal and oral cancer (OAOC). The hypothesis of the researchers is that patients diagnosed with bronchogenic carcinoma have a reduced survival rate if they also have an associated primary OAOC.

## 1. Introduction

Cancer is a leading cause of death worldwide, accounting for nearly 10 million deaths in 2020 [1,2,3]. Each cancer has its own prognosis, which plays a vital role in patient management and clinical decision-making.

Occasionally, different cancer types share risk factors [4,5], leading to the potential occurrence of multiple primary malignancies (MPMs). Second primary lung cancer frequently develops in individuals who have survived head and neck cancer. This occurrence is often attributed to shared risk factors such as tobacco smoking and heavy alcohol consumption [6,7].

Trachea, bronchus and lung cancer, followed by breast neoplasms, were the most common cancers worldwide in 2022, according to the World Cancer Research Fund [8], with an incidence of 2.48 million new cases and causing 1.81 million deaths in that same year [9]. Lung cancer remains a leading cause of cancer mortality in spite of its decline in incidence and mortality in the last decade, accounting for over 20% of cancer deaths annually [10]. On the other hand, oropharyngeal and oral cancer (OAOC) is the 16th most common cancer worldwide, representing approximately 6% of all cancers [6], with an incidence of 389,846 new cases in 2022 and causing 188,438 deaths worldwide in that same year, according to the World Cancer Research Fund [11].

When talking about MPMs, the estimation of their incidence and survival rate is not as simple as in the cases of single primary cancers, since two or more malignancies play a part. Therefore, the incidence rate is quite variable for each organ and has been recently reported to be within a range of 2–17%, which has increased compared to the 4.7% that was initially reported in 1921 [12]. Understanding MPMs requires in-depth knowledge of the cancers that affect each organ separately.

The objective of the present study is to compare the prognosis in terms of survival rate in patients who presented with isolated bronchogenic carcinoma (BC) with those patients who presented with BC and had a personal history of associated OAOC.

## 2. Materials and Methods

An observational longitudinal study with retrospective data collection was conducted, including all consecutive patients who underwent surgical resection with curative intent for BC in the Thoracic Surgery Department of Hospital Clínico San Carlos (HCSC), Madrid, Spain, during the period of December 1989 to December 2024. From this cohort, patients diagnosed with OAOC were identified.

Inclusion criteria:

1. Patients who underwent surgery consecutively with the diagnosis of BC in HCSC.
2. Patients who underwent surgery within the specified period: 1989 to 2024.
3. Patients with BC with an additional diagnosis of OAOC treated with radical purpose.
4. Patients who underwent surgical treatment of their BC with curative intention.

Exclusion criteria:

1. Patients with BC who underwent staging surgery.
2. Patients with BC who underwent diagnostic surgery (biopsy).
3. Patients with BC undergoing surgery with palliative intent.
4. Patients with BC who underwent exploratory thoracotomy (unresectable tumor).
5. Patients with BC who experienced postoperative mortality.
6. Patients with BC diagnosed with another malignancy that was different from oropharyngeal cancer.

The primary outcomes were overall survival and disease-free survival (expressed in months), defined as the time from radical surgery of the lung malignancy to death or to oncological (OAOC or pulmonary) progression. The study covariates were age (expressed in years at the date of surgery), gender (male, female), smoking habit (non-smoking habit, 20 pack-year smoker, 40 pack-year smoker, ex-smoking habit), follow-up (months), and death.

In the descriptive analysis, qualitative variables were summarized by their frequency distribution and quantitative variables were summarized by their mean and standard deviation (±SD). The continuous non-normally distributed variables were summarized by the median and interquartile range (IQR: P25–P75).

Both groups were compared in terms of their baseline demographics and clinical data to identify potential confounding variables. In the case of qualitative variables, comparison was evaluated by the Chi-square test or by Fisher’s exact test if more than 25% of the expected values were less than 5. To compare the two groups, Student’s *t*-test for quantitative normally distributed variables and the non-parametric median test for non-normally distributed variables were used.

Patient survival was estimated using the Kaplan–Meier method for survival tables. The Kaplan–Meier curve displays the probability of survival (in which the event did not occur) as a function of time.

A multivariate Cox proportional hazards regression model was constructed to jointly assess patient survival in each group. The time-to-event variable was adjusted using the intervention date. And the models were reported as hazard ratios (HRs) with 95% confidence intervals (CIs) and *p*-values. The model was also adjusted by baseline variables which were not homogenous among the groups.

Statistical significance was considered at *p* < 0.05.

## 3. Results

**Figure 1:**
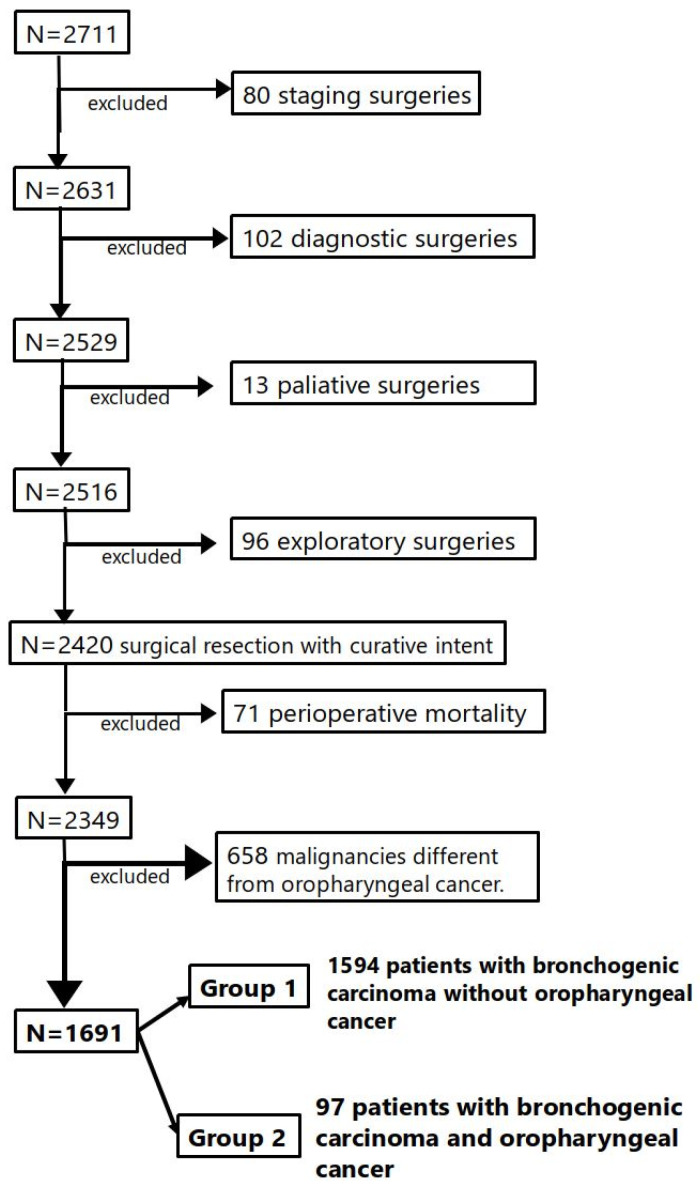
Patients included in the study after the inclusion and exclusion criteria were applied.

After the inclusion and exclusion criteria were applied to our cohort, two study groups were established. From a total of 2711 patients with bronchogenic cancer who underwent surgery, 80 were excluded for undergoing staging surgery, 102 for undergoing diagnostic surgery, 13 for undergoing palliative surgery, and 96 for undergoing exploratory thoracotomy. From the remaining cohort of 2420 patients who underwent surgical treatment with curative intention, 71 experienced postoperative mortality. Of our resulting sample of 2349 patients, 658 were excluded from the study for presenting other second primary malignancies aside from head and neck cancer (Table S1). The final patient sample was formed of 1691 patients who were classified into two groups (Figure 1):

− Group 1: 1594 patients with isolated BC.
− Group 2: 97 patients with BC and previous oropharyngeal cancer.

Regarding the demographic data of the cohort of 1691 patients who underwent radical lung surgery with curative intent in Hospital Clínico San Carlos between 1989 and 2024, the mean age was 64.6 years (SD 9.78) and the median follow-up time was 63 months [IQR 19–111]. The total proportion of males (79.2%) in the study was higher than the proportion of females (20.8%). The median and mean interval between the development of first and second primary cancer were 66.72 [IQR 20.74–104.25] and 75.96 (SD 67.98) months, respectively.

When analyzing both groups, Group 1 was formed of 1594 patients who presented isolated bronchogenic carcinoma, 1248 (78.3%) of which were males while 346 (21.7%) were females. Group 2 was formed of 97 patients who presented BC and history of oropharyngeal cancer, 91 (93.8%) of which were males while 6 (6.2%) were females. The proportion of males was significantly higher (*p* = 0.001) in Group 2 (93.8%) than in Group 1 (78.3%), eliminating gender as a confounding factor when comparing overall survival between groups. There were no significant differences in the mean age between Group 1 (64.5 years, SD 9.91) and Group 2 (66.23, SD 7.23).

There were no significant differences in lung cancer staging at the moment of lung surgery between groups (*p* = 0.14), excluding it as a confounding factor when comparing overall survival. All patients presented a score of 70 or more on the Karnofsky Performance Status Scale and presented a score of 0 or 1 at the time of radical lung surgery.

When comparing smoking ratios in both groups, we found that the proportion of non-smokers, 20-package smokers and 40-package smokers was significantly higher in Group 1 (20.8% non-smokers, 7.7% 20-package smokers and 39% 40-package smokers) versus in Group 2 (6.2% non-smokers, 3.1% 20-package smokers and 36% 40-package smokers). The proportion of ex-smokers was significantly higher in Group 2 (54.6%) compared to Group 1 (32.5%), *p* < 0.001. A smoking habit was excluded as a confounding factor when comparing overall survival between groups. 

The median follow-up in Group 1 was 63.5 months [IQR 19–111.75] and in Group 2 was 49 months [IQR 16–93], with no significant difference between both groups. There were no significant differences in lung cancer recurrence between groups (*p* = 0.122), but mortality was significantly higher in Group 2 (80.4%) compared to Group 1 (66.6%), *p* = 0.02. The characteristics of all the included patients are summarized in Table 1.

### Overall Survival

**Figure 2:**
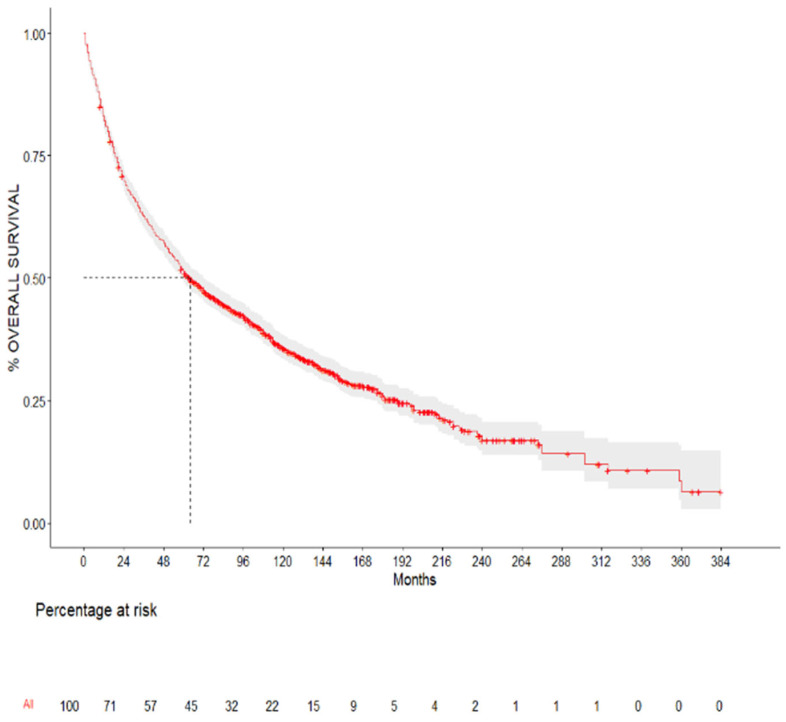
Kaplan–Meier curve for all patients included in the study without group stratification. Median survival: 64 months (CI 95% 59–72 months).

The median survival for all the patients included in the study (N = 1691) was 64 months (CI 95% 59–72 months). The overall survival rates were as follows: 82% at 12 months (CI 95% 80.3–84%), 69.8% at 24 months (CI 95% 67.7–72%), 51.4% at 60 months (CI 95% 49–53.8%), and 35.7% at 120 months (CI 95% 33.4–38.3%). These results are summarized in Figure 2 and Table 2.

**Figure 3:**
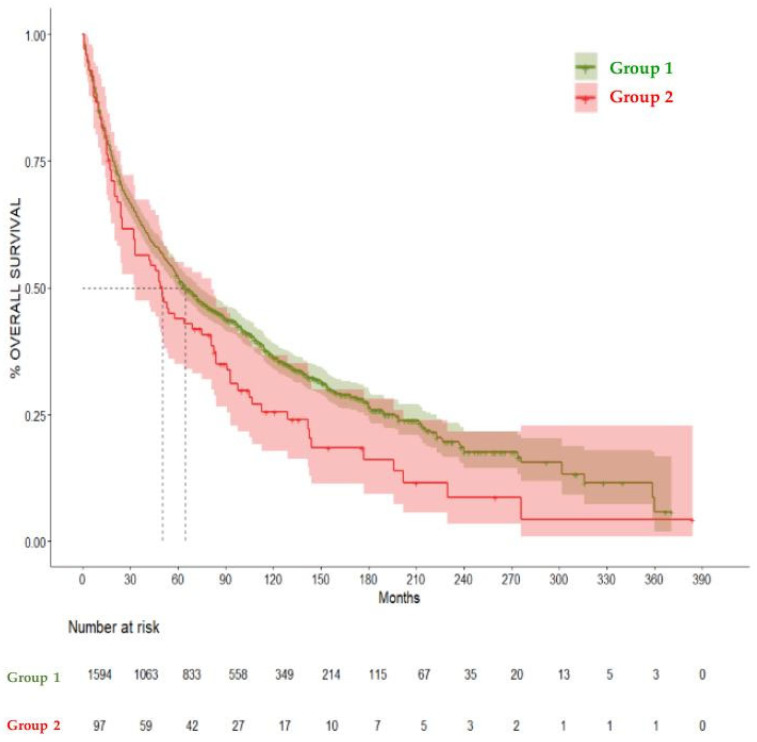
Kaplan–Meier curves comparing overall survival in Group 1 and Group 2. Median Survival for Group 1 was 65 months and for Group 2 was 50 months. (HR) = 1.29, 95% CI = 1.03–1.63, *p* = 0.02.

**Figure 4:**
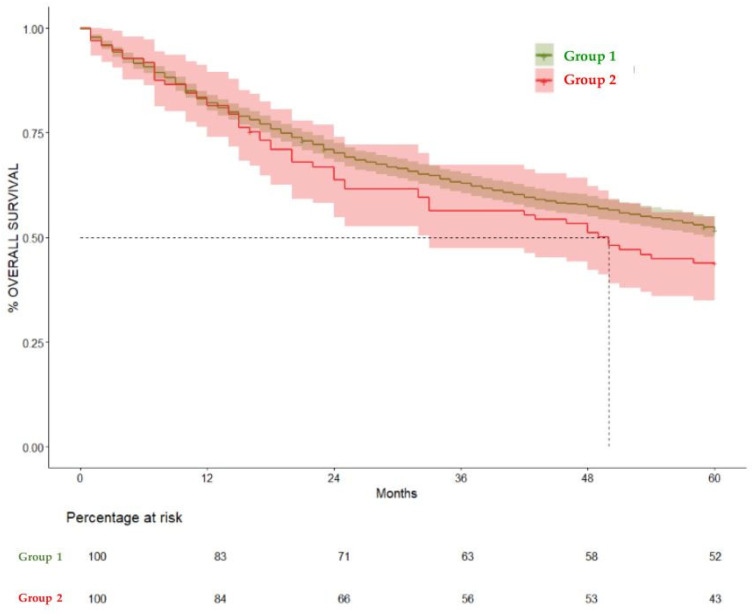
Kaplan–Meier curves comparing overall survival at 60 months in Group 1 and Group 2. HR = 1.23, (CI 95% 0.9–1.6) *p* = 0.14.

**Figure 5:**
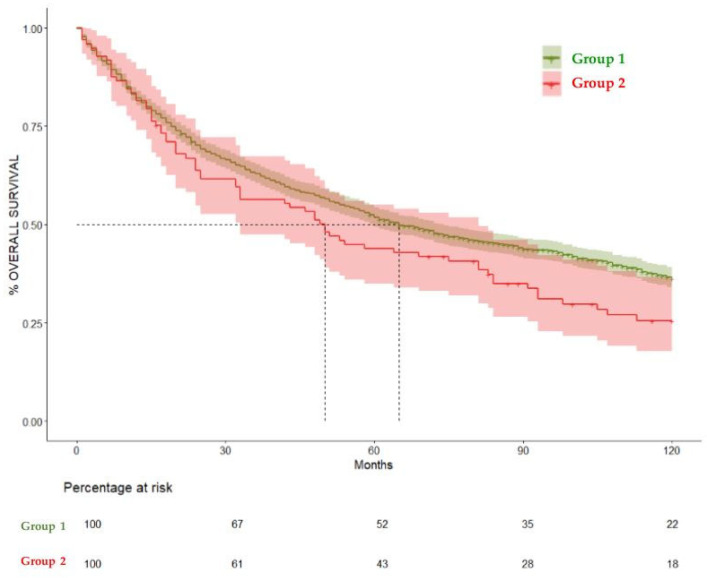
Kaplan–Meier curves comparing overall survival at 120 months in Group 1 and Group 2. HR= 1.28 (CI 95% 1–1.6), *p* = 0.04.

When comparing Group 1 and Group 2, the median overall survival was 65 (CI 95% 60–47) and 50 months (CI 95% 33–81), respectively. Patients who were diagnosed with OAOC (Group 2) had a statistically significant negative effect on overall survival in the total 360-month follow-up (hazard ratio (HR) = 1.29, 95% CI = 1.03–1.63, *p* = 0.02). The negative impact on survival became apparent at 120 months, as Group 2 did not show a significant difference in the 60-month survival rate in comparison to Group 1 (HR = 1.23, CI 95% 0.9–1.6) *p* = 0.14. But when comparing the 120-month survival rate, Group 1 showed a significantly higher survival rate (36.4%, CI 95% 33.9–39%) compared to Group 2, (25.54%, CI 95% 17.78–36.7%) HR= 1.28 (CI 95% 1–1.6), *p* = 0.04. These results are summarized in Table 3, Figure 3, Figure 4 and Figure 5.

There were no significant differences in lung cancer recurrence between groups (*p* = 0.122).

**Figure 6:**
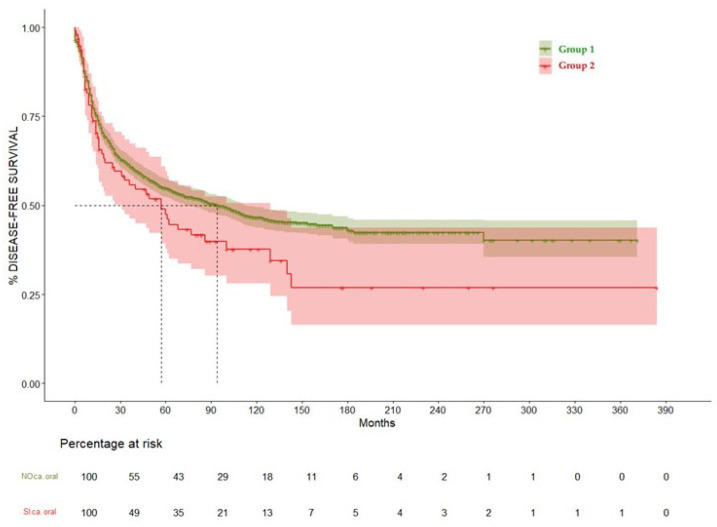
Disease-free survival curve comparing Group 1 and Group 2.

When analyzing disease-free survival (DFS), Group 1 showed median DFS of 94 months (CI 95% 73–112), while for Group 2 it was 57 months (CI 95% 31–129), showing a non-significant trend of higher incidence or oncological relapse among patients with history of OAOC (*p* = 0.06), as we can see in Table 4 and Figure 6.

The median and mean interval between the development of first and second primary cancer were 66.72 [IQR 20.74–104.25] and 75.96 (SD 67.98) months, respectively. The authors included it in the article (line 175).

Regarding disease-specific survival, out of a total of 1139 deaths, 157 were not cancer-related, representing 9% of the total sample of 1691 patients. The reasons for death are listed in Table S2.

## 4. Discussion

A second primary malignancy (SPM) is defined as a histologically distinct malignant tumor that arises in a different anatomical site or tissue and is not attributable to recurrence or metastasis of the first primary malignancy (FPM) [13].

Epidemiological research indicates that the occurrence of multiple primary cancers ranges between 2% and 17% [12]. More specifically, in patients with lung cancer, the incidence of multiple primaries ranges from 13.4% to 22% according to numerous studies [12,14]. Although various researchers have reported a possible correlation between the organ of the second primary cancer and the organ of the first primary cancer, controversy remains [15].

A 2006 analysis of the SEER cancer registry [16] showed that patients with lung cancer had a 5.7% cumulative incidence of developing any second primary malignancy over a 25-year period, with an observed-to-expected ratio (O/E) 1.36, (95% CI = 1.34–1.39) and excess absolute risk (EAR) = 65 per 10,000 person-years. Significantly increased risks were observed for subsequent cancers of the buccal cavity, oropharynx, esophagus, stomach, bowel, pancreas, bladder, kidney, renal pelvis, and leukemia [16]. Similar results were found by Amit Singnurkar et al. [17] in their cohort of early-stage lung cancer patients, as the incidence of multiple primary malignancies was 7.8%, with synchronous tumors accounting for 2.2%. The most frequent sites of synchronous primaries were the gastrointestinal tract, breast, and urinary system.

A more recent study conducted in Thailand by Pariyada Tanjak et al. [18] found that among the top ten most frequent metachronous multiple primary malignancies, head and neck cancer accounted for 11.8%, and lung cancer accounted for 3.2%. Additionally, second primary malignancies tended to occur within 2 years following initial diagnoses of either lung cancer or head and neck cancer. In contrast with the 2006 SEER Registry, this Thai group demonstrated that head and neck cancers had a significant and strong association with the development of second primary lung cancer (RR 2.41; 95% CI: 1.58–3.58), but second head and neck malignancies were not found to be significant after primary lung cancer [18].

A systematic review and meta-analysis by Wang et al. that studied the correlation between second and first primary cancer in 9 million cancer patients [14] showed that although lung cancer had the second highest first primary cancer incidence rate, the incidence rate of second primary malignancies in lung cancer patients was relatively low. More specifically, the incidence rate of second primary head and neck cancer in lung cancer patients was 0.1‰. By contrast, those with a history of primary head and neck cancer developed a second primary lung cancer more frequently.

Numerous studies have identified smoking as a common risk factor for head and neck and lung cancers, among others [4,5]. A population-based study of patients diagnosed with a primary malignancy from the top 10 smoking-related cancer sites by Boakye et al. [16] reported that patients with smoking-related cancers had a 51% increased risk of second primary malignancies (SPMs), being patients with head and neck cancer at the highest risk of developing SPMs. Patients with urinary bladder, kidney, and lung cancers also had increased risks. The oral cavity and pharynx were the most common second primary cancer sites. They are two anatomic regions with their own properties. However, it seems that they may share some epidemiological aspects, and the connection with bronchogenic carcinoma is shared by both. That is why we have included tumors from both regions in the analysis. Human papilloma virus (HPV) is a risk factor that has been shown to play a remarkable role in cancer development, especially in the oropharyngeal region. Its relationship with smoking condition is not clear [19]. On the other hand, our analysis has an important limitation. Our database did not include data about the HPV condition of the patients, so we have not been able to analyze its role in this study. Shiel’s group [20] observed that in comparison to individuals who never smoked, current smokers consuming 20 cigarettes per day exhibited an elevated risk of developing second smoking-related malignancies among survivors of stage I lung cancer (hazard ratio [HR], 3.26; 95% CI, 0.92–11.6), as well as among those with head and neck (HR, 4.45; 95% CI, 2.56–7.73), bladder, and kidney cancers. A notable finding in our study is that the proportion of active smokers is significantly higher among patients with isolated lung cancer (particularly 40-pack smokers), but the proportion of former smokers among lung cancer patients with OAOC history is significantly higher compared to those with isolated lung cancer. These findings suggest that patients who had one of these cancers in the past decided to discontinue smoking but eventually developed a second primary cancer, indicating that the detrimental effect of smoking remains present even after ceasing the smoking habit.

Current evidence regarding the prognostic implications of second primary tumors in lung cancer remains limited and inconsistent [18]. In a study by Duchateau et al. [21], patients with non-small cell lung cancer who developed second primary tumors demonstrated improved overall survival compared to those without such tumors. In contrast, our study shows that a personal history of OAOC in lung cancer patients was associated with an increased risk of mortality, as patients with both oropharyngeal and lung cancer (Group 1) exhibited a 1.29-fold higher risk of death compared to those with isolated lung cancer (Group 2). These findings highlight the potential impact of a prior oral cancer diagnosis on the prognosis of patients with lung cancer, emphasizing the need for comprehensive evaluation and monitoring in this patient population. The fact that the median interval between the development of first and second primary cancer surpasses the boundary of five years that is usually recommended as time of follow-up for patients undergoing radical treatment of either head and neck or pulmonary carcinoma (66.72 months) suggests that despite sharing risk factors, they are two differentiated oncological processes.

Moreover, a Chinese study carried out by Feng Li et al. [22] demonstrated that in lung cancer patients with multiple primary malignancies, those with metachronous malignancies exhibited significant superior overall survival (OS) 72.8 (range 12.2–391.0) compared to patients with synchronous malignancies OS = 12.9 (range 0.8–86.3) months, respectively. Amit Singnurkar’s group [17] also studied the impact of synchronous malignancies on survival in patients with early-stage curable non-small-cell lung cancer. They found that the diagnosis of a synchronous malignancy within 180 days of lung cancer onset was associated with a significantly worse overall survival, with a hazard ratio (HR) of 1.45 (95% CI: 1.17–1.80). Among patients with stage I and II lung cancer, the 5-year survival rates were 31.3% and 39.2% for those with synchronous malignancies, compared to 56.2% and 39.4% for those without. Although our study showed no significant differences in cumulative survival at 60 months between patients with isolated lung cancer and those with personal history of oropharyngeal cancer, a marked reduction in survival was evident in the latter group at 120 months. This suggests that the coexistence of a personal history of OAOC may negatively influence the long-term prognosis of patients with lung cancer, particularly beyond the 10-year mark. Moreover, interestingly, disease-free survival showed a non-significant trend of being worse among patients with a history of OAOC, even within the first 5 years of follow-up. These findings underscore the importance of considering prior oncological history when assessing survival outcomes and planning long-term follow-up strategies. Early detection of those relapsed probably led to an optimal therapeutic approach in so far as patients were treated with radical purpose, although this topic has not been analyzed. It has been suggested that patients referred to long-term survivors after suffering a neoplastic process may represent a select cohort with specific oncological issues that deserve an individualized deal [23].

By contrast, in another Chinese retrospective study of lung cancer patients performed by Lu et al. [24], those with a secondary malignancy (9.5%) showed significantly better median survival (19.09 vs. 9.53 months; HR 0.66, 95% CI: 0.55–0.79) compared to patients without one. Survival varied based on the timing of the second cancer: patients who developed a secondary malignancy during follow-up had the best outcomes compared to those who had pre-existing malignancy and synchronous malignancies. These findings suggest that timing of secondary malignancy influences prognosis in lung cancer patients.

A study by Hyuna Sung et al. [25] reported that among 5-year survivors of lung cancer, the incidence of second primary malignancies was 223 and 144 per 10,000 person-years in men and women, respectively, with corresponding relative risks of 1.20 (95% CI: 1.16–1.26) and 1.17 (95% CI: 1.12–1.23). Additionally, mortality from oral cavity and pharynx secondary primary cancers in this population was 3.3 and 1.1 per 10,000 person-years in men and women, with standardized mortality ratios (SMRs) of 2.08 (95% CI: 1.46–2.88) and 1.8 (95% CI: 1.01–2.97), respectively. These findings indicate that lung cancer survivors, especially men, have a significantly increased risk of developing and dying from second primary cancers, including oral cavity and pharynx cancers, compared to the general population. Similarly, in our study, the proportion of male patients (93.8%) with lung cancer and personal history of oropharyngeal carcinoma was significantly higher than that of female patients (6.2%) within the same cohort, suggesting an increased risk among males to develop second primary malignancies.

## 5. Conclusions

In our cohort, a smoking habit and masculine gender were found to be a risk factor for developing both lung and oropharyngeal cancers. Lung cancer patients who presented with a personal history of OAOC had worse overall survival compared to patients who presented isolated lung cancer, and significant differences were found at 120 months of follow-up.

## Figures and Tables

**Table 1:** Characteristics of all included patients, total and by groups. Group 1: isolated lung cancer. Group 2: lung cancer + oropharyngeal cancer.

		Total (N = 1691)	Group 1 (N = 1594)	Group 2 (N = 97)	*p* Value
Mean Age (SD)		64.4 (9.78)	64.55 (9.91)	66.23 (7.23)	0.16
Gender (%)	Male	1339 (79.2%)	1248 (78.3%)	91 (93.8%)	0.001
	Female	352 (20.8%)	346 (21.7%)	6 (6.2%)	
Lung cancer staging	I	838 (51.2%)	781 (50.6%)	57 (61.3%)	0.14
	II	345 (21.1%)	336 (21.7%)	9 (9.7%)	
	III	415 (25.3%)	389 (25.2%)	26 (28.0%)	
	IV	40 (2.4%)	39 (2.5%)	1 (1.1%)	
Smoking habit (%)	No	320 (19.9%)	314 (20.8%)	6 (6.2%)	<0.001
	20 PY	120 (7.5%)	117 (7.7%)	3 (3.1%)	
	40 PY	624 (38.8%)	589 (39.0%)	35 (36.1%)	
	Ex-smoker	544 (33.8%)	491 (32.5%)	53 (54.6%)	
Mean follow-up [IQR]		63 [19–111]	63.5 [19–111.75]	49 [16–93]	0.4
Exitus (%)		1139 (67.4%)	1061 (66.6%)	78 (80.4%)	0.02

**Table 2:** Overall survival of total patients without group stratification.

Months	N at Risk	N Event	Survival	Lower 95% CI	Upper 95% CI
12	1407	146	82.1%	80.3%	84%
24	1194	208	69.8%	67.7%	72%
60	875	69	51.4%	49%	53.8%
120	366	34	35.7%	33.4%	38.3%

**Table 3:** Overall survival (%) expressed in months comparing Group 1 and Group 2.

Months	%Survival Group 1 (95% CI)	%Survival Group 2 (95% CI)	*p* Value
12	82.2% (80.3–84.1)	81.44% (74.06–89.6)	0.1
24	70.2% (68–72.5)	63.76% (54.85–74.1)	0.1
60	51.8% (49.4–54.3)	43.9% (35.02–55)	0.141
120	36.4% (33.9–39)	25.54% (17.78–36.7)	0.0466

**Table 4:** Comparison of disease-free survival (DFS) between groups, expressed in months.

Months	DFS Group 1 (95% CI)	DFS Group 2 (95% CI)	*p* Value
12	77.2% (75.1–79.4%)	73.7% (65.2–83.3%)	0.06
24	66.6% (64.2–69.1%)	62% (52.6–73%)	
60	54.8% (52.2–57.4%)	47.5% (37.8–59.6%)	
120	46.5% (43.8–49.4%)	37.7% (28–50.7%)	

## Data Availability

The data presented in this study are available in this article and Supplementary Materials.

## Supplementary Materials

The following supporting information can be downloaded at https://www.mdpi.com/article/10.3390/cancers17111850/s1, Table S1: Cancer types we found in our study (N = 658); Table S2: Specific reasons of mortality during the follow-up.
