# Supplementary material for: Oropharyngeal and Oral Cancer in Lung Cancer Patients: Do They Present a Worse Prognosis than Isolated Lung Cancer Patients?
Source: Cancers (Basel). 2025 May 31;17(11):1850. doi: 10.3390/cancers17111850 (PMC12153655; doi:10.3390/cancers17111850)
Supplement: Supplementary file 1 [file cancers-17-01850-s001.zip › cancers-3648025-supplementary.pdf]

Table S1. Cancer types we found in our study (N = 658).

Other Cancer types	N (%)
Prostate carcinoma	92 (13.98%)
Colon carcinoma	85 (12,91%)
Urothelioma	81 (12.3%)
Breast	75 (11.41%)
Melanoma	37 (5.62%)
Rectum carcinoma	30 (4.55%)
Lymphoma	30 (4.55%)
Kidney	28 (4.26%)
Hepatocellular carcinoma	20 (3.04)
Meningioma	19 (2.89%)
Gastric carcinoma	19 (2.89%)
Leuchemia	18 (2.73%)
Sarcoma	16 (2.43%)
Cervix carcinoma	11 (1.67%)
Endometrium carcinoma	11 (1.67%)
Plasmocytoma	10 (1.52%)
Ovary	9 (1.37%)
Esophagus carcinoma	8 (1.22%)
Pancreatic carcinoma	8 (1.22%)
Thyroid	8 (1.22%)
Anus	6 (0.91%)
Cholangiocarcinoma	6 (0.91%)
Myeloproliferative syndrome	6 (0.91)
Appendix carcinoid	5 (0.76%)
Pheocromocytoma	5 (0.76%)
Seminoma	5 (0,76%)
Hypophysis adenoma	4 0.61%)
Vulval	4 (0.61%)
Thymoma	2 (0.3%)

Table S2. Specific reasons of mortality during the follow-up.

Cause	N
Sepsis/ infections	74
Cardiovascular	40
Pulmonary embolism	15
COVID-19	8
Encephalopathy	8
Bronch aspiration	7
Stroke	2
Respiratory failure	2
Traffic accident	1
